# Frequency of Genital Involvement in Women with Oral Lichen Planus in Southern Iran

**DOI:** 10.1155/2012/365230

**Published:** 2012-05-23

**Authors:** M. Davarmanesh, A. Samsami Dehaghani, Z. Deilami, A. Monabbati, L. Dastgheib

**Affiliations:** ^1^Department of Oral Medicine and Diagnosis, School of Dentistry, Shiraz University of Medical Sciences, Ghasrodasht Avenue, 71956-15878 Shiraz, Iran; ^2^Department of Obstetrics and Gynecology, School of Medicine, Shiraz University of Medical Sciences, Zand Avenue, Imam Hossein Square, P.O. Box 71436-66184 Shiraz, Iran; ^3^Department of Pathology, School of Medicine, Shiraz University of Medical Sciences, Zand Avenue, Imam Hossein Square, P.O. Box 71436-66184 Shiraz, Iran; ^4^Department of Dermatology, Shiraz Skin Research Center (SSRC), School of Medicine, Shiraz University of Medical Sciences, Zand Avenue, Imam Hossein Square, P.O. Box 71436-66184 Shiraz, Iran

## Abstract

*Background*. Lichen Planus is a chronic mucocutaneous disease of immunological basis and unknown etiology. women with oral lichen planus may have concomitant manifestations in vulvovaginal areas. *Objective*. To determine the frequency and risk factors of genital involvement in a group of Iranian women affected by oral lichen planus. *Methods*. Thirty-six women with clinical and histopathological diagnosis of oral lichen planus were evaluated for demographic, historical, and clinical parameters of the oral disease. All the patients were referred for careful vulvovaginal examination, as well as histopathological assessment upon clinical indication. *Results*. Nineteen patients complained from genital symptoms but the number of women with the final diagnosis of genital lichen planus (*n* = 2) was too small to show any correlation with the parameters evaluated. *Conclusion*. In spite of low genital involvement possibly due to inadequate patient population, lack of follow-up visits, and contribution of genetic or ethnic factors, for conservative patient care, women with the oral lichen planus in particular those having some relevant genital symptoms, should preferably be referred for careful vulvovaginal examination. Multicenter cohort studies on women of different geographical regions or ethnicities who have genital lichen planus alone or in combination with other common sites are encouraged.

## 1. Introduction

Lichen Planus (LP) is an inflammatory disease of the stratified squamous epithelia with an unknown etiology [1]. LP may involve mucosal surfaces such as the oral, genital, and other mucosae and the skin including the scalp and nails [2]. It affects most frequently the oral mucosa with the estimated prevalence between 0.5–3%, female/male sex ratio ranging between 1.5–3, the age of onset generally between 30 and 60 years and a premalignant potential under much debate [1]. Oral lichen planus (OLP) may manifest in six clinical forms individually or combined: papular, reticular, plaque-like, atrophic, erosive, and bullous. The lesions are chronic, rarely undergo spontaneous remission, and are often a source of morbidity [3].

Patients with OLP frequently have concomitant manifestations in one or more extraoral sites [3]. The association between LP of the vulva, vagina, and gingiva was firstly recognized and defined as vulvovaginal-gingival syndrome [4], or plurimucosal LP [5] which thereafter was commonly called female genital LP (GLP). It may occur as a classic form with typical papules on the perigenital skin (labia majora) [6] and asymptomatic reticular lesions [7], or affect the mucosal side of the vulva which is typically manifested as a glazed erythema with possible development of secondary erosions [6]. Various symptoms including burning, pain, vaginal discharge, and dyspareunia are frequent in patients with erythematous and erosive disease [3]. Moreover, there are the sequelae of vulval scarring and vaginal stenosis that affect sexual function [8], and may pose a small but definite risk of malignant change [8–12].

 In addition to articles delineating clinical characteristics of the vulvovaginal-gingival syndrome [2, 7, 13–16], a few descriptive studies in looking for possible association between LP of oral versus female genital areas have reported the incidence of GLP among OLP women varying between 19–58% [17–19].

OLP is one of the most common reasons for patients' referral to our Oral Medicine Department, especially in women. To the best of our knowledge, there has been no report in the English language literature to give an estimate on the frequency of GLP in Iranian women having OLP. Therefore, we decided to perform this study to investigate the frequency of genital involvement expected among Iranian women with OLP and the possible association between demographic, clinical, or histological features of OLP and a higher risk of genital involvement.

## 2. Patients and Methods

The proposal of the study was firstly reviewed and approved by the Research Council of Shiraz Dental School, Shiraz, Iran. Fifty women who referred consecutively to the Oral Medicine Clinics of Shiraz Dental School, Shiraz, Iran, from Spring 2007 to Autumn 2009 with a preliminary clinical diagnosis of OLP were selected to participate in our study.

The exclusion criteria were as follows: (1) any use of systemic medications with potential suppressive effect on LP disease for at least a period of 6 months before examination; (2) patients showing any lichen planus-like lesion in a mucosal site in close contact or vicinity of possible causative dental restorations particularly amalgam (naturally such a local factor denies an etiologic role to cause the disease in a distant site such as genitalia); (3) any history of jaundice, or any family or occupational history that might place the patients at risk of hepatitis C infection.

The study process and its objectives were explained to all patients, and informed consents were obtained. Then, the demographic, historical, and clinical data related to OLP were recorded. During the oral examination sessions, the patients were questioned regarding the possible existence of pruritic cutaneous lesions of LP, as well as any of the genital symptoms of pain, burning or pruritus, discharge, and dyspareunia. Then, incisional biopsy samples were taken from the patients from a representative part of the oral lesions' margin by one of the authors. All patients with histopathological diagnosis of OLP, regardless of having genital symptoms or not, were referred for examination of the vulvovaginal and cutaneous areas to Shahid Faghihi Polyclinic, Shiraz University of Medical Sciences, within one week after oral examination. At the clinic, careful vulvovaginal examination of each referred patient was performed by the collaboration of two authors specialized in fields of gynecology and dermatology. At the same appointment, clinical assessment of the relevant cutaneous lesions of each patient was performed by the dermatologist. During the genital examination, an incisional biopsy sample was taken with the patient's consent by the gynecologist if there were any abnormality in vulval or vaginal sites which needed histopathological diagnosis regardless of being related to LP or not.

In this study the modified World Health Organization's (WHO) diagnostic criteria of OLP proposed by van der Meij and colleagues [20] were used for diagnostic classification of patients' oral disease as well as histological diagnosis of GLP.

All data were analyzed by SPSS software, version 11.5. Fisher's exact test was used for comparison of the ratio of GLP occurrences in the OLP patients of our cross-sectional study and that of another study [19] with the same design. *P* values < 0.05 were statistically significant.

## 3. Results

**Figure 1:**
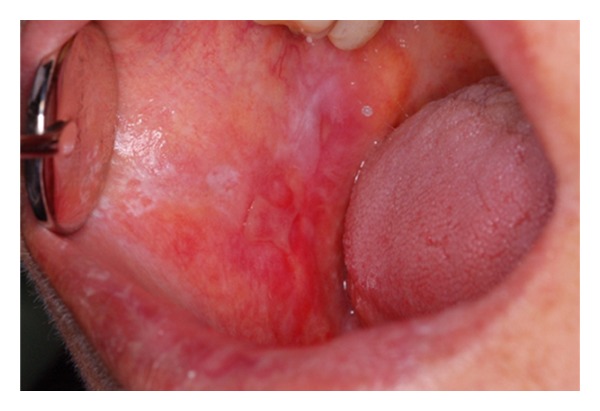
Clinical features of oral lichen planus manifested as keratotic, atrophic, and erosive components in buccal mucosa.

Of the 50 women who initially enrolled in the study, 14 women refused to attend the gynecological examination for personal reasons. In all the remaining 36 women, the set of histopathological features of the mentioned diagnostic criteria [20] were completely fulfilled by the microscopic findings of the patients' lesions (Figure 1). On the part of the clinical criteria, the features of six patients were not completely fulfilled; however, because histological findings of the six, as of the other patients were completely fulfilling, we called all the patients collectively under the same diagnostic category of OLP. Frequencies of various combinations in the clinical types of oral lesions among 36 OLP patients are shown in Table 1.

The patients' ages ranged between 29 and 65 with a mean of 49 years. The approximate duration from the first presentation of the oral disease to our oral examination session ranged from 7 days to 14 years. Upon clinical oral examination, 24 patients (67%) complained about persistent oral pain and discomfort at rest or when eating some kinds of spicy or hot foods.

Review of all the patients' records revealed that 11 (31%) patients had recently used some medications which were among the list of medications with known potential to cause lichenoid or lichen planus-like drug eruptions (LDErs) [21]. Eight (22%) patients had a history of pruritic cutaneous lesions, which had resolved either spontaneously or by medication, but dermatological examination showed that four (11%) patients had cutaneous LP concurrent with OLP. The distribution of patients per disease and category of systemic medications used on a regular basis at the time of initial clinical examination were shown in Table 2. Also included in the table is the row representing the medications bearing the potential to cause LDEr and their number of uses in the study patients.

**Figure 2:**
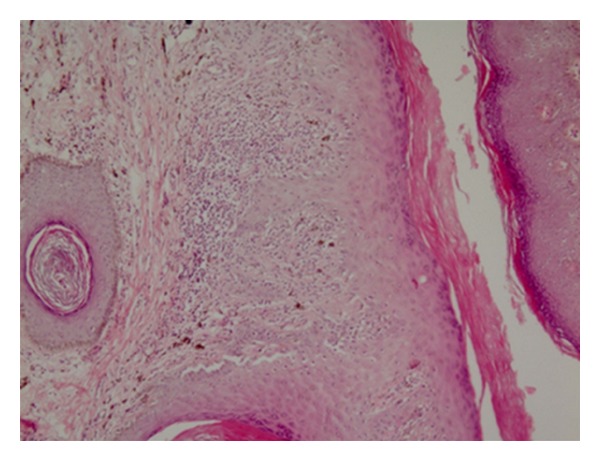
Photomicrograph (×40) of genital lichen planus with fulfillment of diagnostic histopathological criteria.

**Figure 3:**
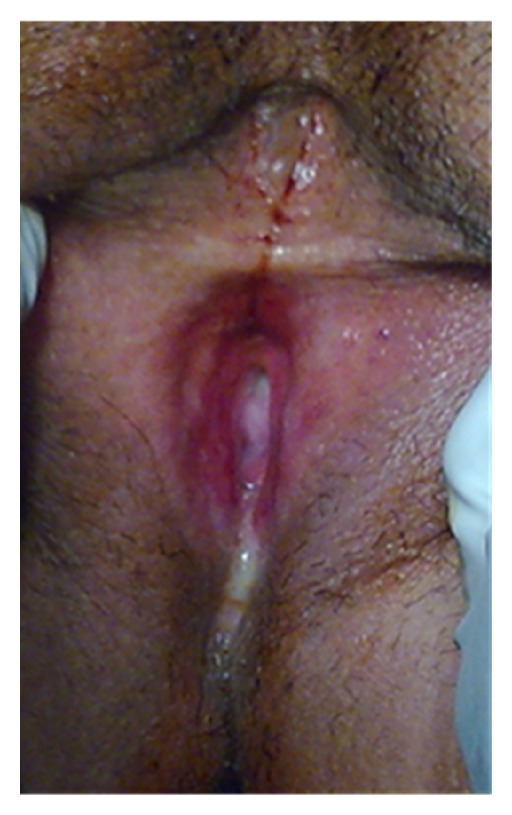
Vulvar lichen planus manifested as glazed erythema and erosion of inner aspect of labia majora and labia minora around the vaginal orifice with associated atrophy, scarring, synechia, and stenosis of vagina leading to pain and dyspareunia.

Among the 36 OLP patients, the genital-related symptoms of burning or pruritus, pain, discharge, and dyspareunia were found in 19 (53%) patients either individually or in combination. In the gynecological examination of the vulvovaginal sites, clinical abnormalities were seen in four patients for whom either excisional or incisional biopsy samples from a representative site of the lesions' margin were taken. In one patient, a nonspecific thickening of skin with no clinical suspicion of LP was seen, which was microscopically diagnosed as a retention cyst. In another patient whose clinical examination showed vaginal erythema and erosion, the evaluation of the biopsy sample showed a nonspecific inflammation. However, for the other two patients, as shown in Figure 2, the diagnoses of GLP were made based on definite histopathological criteria [20]. In both of these patients, vulval atrophy and keratosis were clinically seen, accompanied by vulval erosion and vaginal stenosis in one patient who had experienced a long duration of oral as well as genital involvements for about 14 years (Figure 3). As shown in Table 3, because of the low number of the patients having GLP, we were unable to perform any analysis of the recorded parameters to estimate reliably the risk of the GLP occurrence.

## 4. Discussion

In this cross-sectional study, among a group of 36 women with OLP who were referred for vulvovaginal examination, genital lichen planus was diagnosed in two (6%) patients based on both clinical and histopathological features. There were no clinical abnormalities in the genital area in 32 (89%) patients.

This finding is considerably different from the results of studies done in other countries with similar aims and objectives. Eisen and colleagues [17] evaluated 399 women with OLP over a mean period of four years and found genital lichen planus in 77 (19%) patients only by clinical examination. Belfiore and coworkers [18] studied 42 women with OLP over a period of three years. They used clinical and histological examinations and reported a genital involvement in 24 (57%) patients. It was not exactly stated how many times the patients in those studies underwent gynecological examinations for the incidences reported. However in our study the inadequate number of the OLP patients and the lack of gynecologic follow-up visits in the study period can potentially weaken the reliability of comparisons between the results of those studies and of ours. It can be assumed that if our patients' genital examinations were repeated over a longer period of time, more patients with GLP could be found. Such an assumption is consistent with a retrospective review on the characteristics of 122 patients with vulvovaginal-gingival syndrome, which reported the nonsynchronous involvement of the oral and genital sites in 56.3% of patients [2]. 

 Di Fede and colleagues [19] in a cross-sectional study on a group of 41 OLP women which is comparable to our sample size, reported the histopathologically proven GLP in a statistically greater proportion of patients (24/41 = 58%) than our study (*P* < 0.001). It is noteworthy that on a theoretical basis, potential waxes and wanes in the involvement of the two different sites during a chronic disease can possibly deter researchers from a true estimation on the relationship between their occurrences. This feature has been pointed out on the natural course of OLP in a population-based study [22], as more or less is seen during our daily follow-up examinations in some men or women with OLP. Whether such the well-known waxes and wanes in course of the oral lichen planus occur in the lichen planus of vulvovaginal areas, needs to be investigated. It seems that cross-sectional studies cannot give a valid and reliable estimate on the association between LP of oral and genital areas even within a given population of patients. However besides the factors mentioned in explaining such the great disparities between the genital involvements of the OLP women in our study and those of the other comparable studies [17–19], there still might be other important reasons.

Among the main factors responsible for the complex etiopathogenesis of OLP [23], the role of genetic factors on the development of some clinical subtypes of LP have been proposed by several studies. The chance of hepatitis C-associated OLP in patients of some geographic regions [1], the role of genetic polymorphism of two cytokines (IFN-*γ* and TNF-*α*) in partial determination of the oral versus orocutaneous sites of LP development [1], and a possible association between the class II human leukocyte antigen DQB1*0201 allele and development of a severe variant of vulvovaginal-gingival syndrome [15] are among the proposed genetic factors. Hypothetically, it can also be assumed that the genetic background of diverse populations examined in different studies along with the possible contribution of some yet unknown disease modifying factors could manifest GLP in a variety of frequencies and severities, regardless of being associated with OLP or not. Establishment of genetic studies on the GLP patients of different ethnicities or geographic regions can potentially clarify the role of genetic background of the patients in development of genital involvement in the context of either OLP patients or those having LP in the other commonly involved sites.

In our study, genital complaints were not limited to two patients with definite GLP, but the symptoms were also recorded in 17 patients without GLP on genital examinations. This high rate of genital symptoms could partly be attributed either to atrophic mucosal changes related to menopausal states, or some common inflammatory genital conditions including fungal infection. In the case of oral mucosal LP, effects of the superimposing candidal infection and its potential to modify the mucosal surface features of lichen planus, or roles of antifungal treatment in turning the lesion's altered features back to those typical of LP or in partial resolution of OLP and its symptoms have been reported previously [24, 25]. In this study, no attempts in looking for possible causes of the patients genital symptoms were planned. Whether the possible role of candidal infection in our patients' external genitalia, as of oral mucosa could have affected the clinical features of a weak preexisting GLP, or have altered the disease recognition or its presenting symptoms, needs further investigations on the pathogenesis of GLP.

The abundance of genital symptoms besides the low incidence of definite GLP which were also symptomatic differs largely from the results of previous studies which have reported higher frequencies of GLP, with a significant minority of the patients having no genital symptoms [17–19]. Such disparate results confirm the fact that any association between the occurrences of lichen planus in the two mucosal sites could not be so straightforward as it initially seemed, since there may be great complexities and variations in factors responsible for the disease development and the way it manifests in the two anatomically distinct mucosal sites. Although our study was performed on a limited group of OLP women and yielded a low rate of but symptomatic genital involvement, considering even a very low possibility for the development of SCC in the background of GLP [8–12], a simple conclusion will advise the gynecologic examination preferably for the OLP women who complain from genital symptoms. However noting the asymptomatic cases in a considerable proportion of GLP patients in the other comparable studies [17–19], our study with its limitations and the small number of symptomatic GLP cannot reliably deny possible existence of symptomless cases of GLP in our community. Therefore for conservative patient care and until attaining a more reliable data on the genital symptom profile of the Iranian women with GLP, it seems better to refer all OLP women for gynecological examination, regardless of the existence of genital symptoms. However, this approach needs great attempts on the side of clinicians such as oral medicine specialists or dermatologists to inform the LP patients about their condition, course, and possible susceptibility to asymptomatic genital involvement, in order to motivate them for proper referral and examination. Gynecologists should also be able to recognize the manifestations of GLP in close cooperation with dermatologists and to motivate the patients for frequent appointments for management and followup.

In this study, we confronted the unfortunate exclusion of 14 OLP women who refused the gynecologic referral for any reason. Some important factors may have contributed to this noticeable fraction of undesirable patient exclusion from the study such as difficulties in patients' referrals to distant locations in need for various clinical examinations to be performed by different experts and some cultural constraints on examination of the genital sites or organs, which vary greatly in different people. Since in our study the OLP patients sample size was not large enough, and the number of patients with definite GLP was too small, we were unable to perform any analysis of the recorded parameters to estimate reliably the risk of the GLP occurrence. Multicentral and long-term cohorts in patients of OLP or LP of other common sites in different geographical regions will lead to a better understanding of the female GLP natural course and promote earlier disease detection with a greater success in the prevention of complications such as malignancy.

## Figures and Tables

**Table 1:** Frequency of clinical types of oral lesions in the OLP patients.

Clinical type of OLP	Number of patients (%)
Reticular	4 (11.1)
Atrophic	1 (2.8)
Reticular + atrophic	19 (52.8)
Reticular + atrophic + erosive/ulcerative	12 (33.3)

	Total = 36 (100%)

OLP: oral lichen planus.

**Table 2:** Diseases and systemic medications used on a regular basis among 36 OLP women at the initial clinical examination.

Systemic disease or condition	Number of cases
Diabetes mellitus	4
Hypothyroidism	4
Mitral valve prolapse or palpitation	4
Arterial hypertension	3
Allergic disorders	2
Rheumatoid arthritis	1
Osteoporosis	1

Medication category	Number of uses in each category

Antihypertensives	8
Hormones	5
Oral hypoglycemic agents	4
Vitamin + mineral	4
NSAIDs	3
Antidepressants	3
Lipid-regulating agents	3
Insulin	2
Proton pump inhibitors	2
Bisphosphonates	1
Minor tranquilizers	1
Drugs with potential to cause LDEr*	12**

OLP: oral lichen planus; NSAIDs: nonsteroidal anti-inflammatory drugs; LDEr: lichenoid drug eruption.

*An etiologic category of lichen planus caused by use of systemic medications.

**Isolated medications with number of uses inside parentheses in 11 patients: propranolol (5), captopril (1), NSAIDs (4), and metformin (2).

**Table 3:** Distribution of historical and clinical parameters in OLP patients based on having definite GLP or not.

Parameter or condition	Number of patients (%)	
	with GLP *n* = 2	without GLP *n* = 34
Oral symptom	2 (100)	22 (64.7)
Genital symptom	2 (100)	17 (50)
History of cutaneous LP	1 (50)	7 (20.5)
Concomitant cutaneous LP	1 (50)	3 (8.8)
Recent use of drugs with LDEr potential	0 (0)	11 (32.3)

Oral surfaces involved		

Lips	2 (100)	8 (23.5)
Labial mucosa	0 (0)	7 (20.5)
Gingival or alveolar mucosa	2 (100)	28 (82.4)
Buccal mucosa	2 (100)	27 (79.4)
Hard palate	1 (50)	7 (20.5)
Soft palate	0 (0)	1 (3)
Dorsal tongue	1 (50)	2 (6)
Ventrolateral tongue or floor of the mouth	0 (0)	10 (29.4)

OLP: oral lichen planus; GLP: genital lichen planus; LP: lichen planus; LDEr: lichenoid drug eruption.
